# Supplementary material for: Non-Syndromic and Syndromic Defects in Children with Extracranial Germ Cell Tumors: Data of 2610 Children Registered with the German MAKEI 96/MAHO 98 Registry Compared to the General Population
Source: Cancers (Basel). 2024 Jun 6;16(11):2157. doi: 10.3390/cancers16112157 (PMC11172205; doi:10.3390/cancers16112157)
Supplement: Supplementary file 1 [file cancers-16-02157-s001.zip › cancers-2996578-supplementary.pdf]

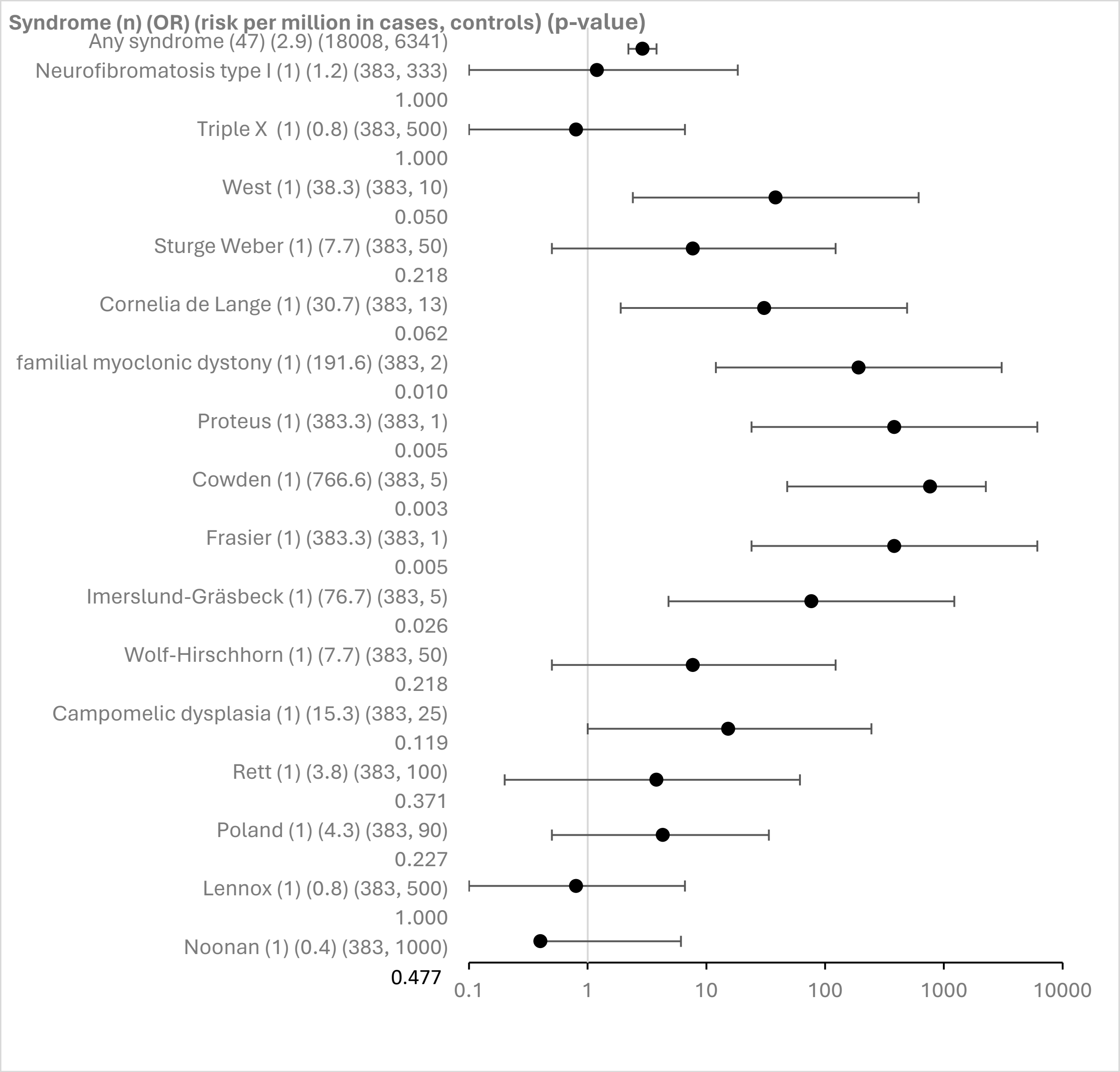

Supplementary Figure S1. Odds ratio and 95% confidence intervals for eGCT in patients with syndromes
